# Editorial: Immunological imbalance: What is its role in intervertebral disc degeneration?

**DOI:** 10.3389/fcell.2023.1196377

**Published:** 2023-04-20

**Authors:** Benjamin Gantenbein, Zhen Sun, Zhongyang Liu, Dino Samartzis

**Affiliations:** ^1^ Tissue Engineering for Orthopaedics and Mechanobiology, Bone and Joint Program, Department for BioMedical Research (DBMR) of the Medical Faculty, University of Bern, Bern, Switzerland; ^2^ Department of Orthopaedic Surgery and Traumatology, Inselspital, Bern University Hospital, Medical Faculty, University of Bern, Bern, Switzerland; ^3^ Department of Orthopaedics, Xijing Hospital, Fourth Military Medical University, Xi’an, China; ^4^ Department of Orthopaedics, Chinese PLA General Hospital, Beijing, China; ^5^ Rush Medical College, Rush University, Chicago, IL, United States

**Keywords:** miRNA, single-cell RNA-seq, intervertebral disc, IL17, immune cells, macrophages, low back pain

## 1 Introduction

Low back pain (LBP) is a major cause of disability worldwide (GBD, 2017 Disease and Injury Incidence and Prevalence Collaborators, 2018) and belongs to the most urgent priorities to identify novel therapies, especially for the elderly (Teichtahl et al., 2015; Chen et al., 2020; Lee et al., 2021). Global health costs are generally rising but in the field of orthopedics and mostly for spine, the costs are exploding in recent years compared to other diseases (Wieser et al., 2011; Martin et al., 2019). Surgical options to treat LBP efficiently are very limited; clinical outcome is often non-satisfactory for the patients with very high re-operation rates (Nachemson et al., 1996; Knezevic et al., 2021). In clinical daily life most of the time “discectomy” is practiced, which is the surgical removal of the painful intervertebral disc (IVD) with a subsequent insertion of a metallic cage as a placeholder and pedicle screws and rods to achieve a so-called spinal fusion (Martin et al., 2019). LBP has many facets but in the cases where the pain is caused from degenerated IVDs, it is usually caused from a reduction in disc height, which then leads to continuous irritation of the nerves. Traditionally, there were mainly two hypotheses formulated that IVDs degenerate early apart from genetic pre-disposal. The first one is the so-called “limited nutrition hypothesis” formulated by Jill Urban and colleagues (Urban et al., 2004; Huang et al., 2014). The second hypothesis was formulated by Adams and Roughley (2006) that claims that IVDs can degenerate because of mechanical overloading. These two hypotheses do not exclude themselves. Possibly in reality, it is often a combination of both, mechanical overloading and limited nutrition (Zengerle et al., 2021). Of course, it is said from these perspectives that the immune-system does not play a major role for IVD degeneration as it was always claimed that the lack of blood vessels in the healthy IVD makes this an “immune-privileged” environment (Bermudez-Lekerika et al.). However, there is increasing evidence that the immune-system might also play a central role in the onset of IVD-induced LBP (Lambrechts et al., 2023). Also the importance of bacteria entering the IVD through the endplates or through other routes, e.g., through the outer annulus fibrosus (AF), were recently discussed (Dudli et al., 2018; Bermudez-Lekerika et al.; Ye et al., 2022). It has been hypothesized that bacterial infection might be a possible cause for so-called type 1 Modic changes as revealed by magnetic resonance imaging (MRI) (Modic et al., 1988; Dudli et al., 2018).

This Research Topic entitled “Immunological Imbalance: What is its Role in Intervertebral Disc (IVD) Degeneration?” focuses on current insights of possible causes for IVD degeneration in the context of the neglected role of the microbiome (Steves et al., 2016; Jackson et al., 2018; Rajasekaran et al., 2020). The Research Topic comprises a total of five publications, whereas three of these are reviews articles (Li et al.; Bermudez-Lekerika et al.; Suyama et al.) and two of them are original research articles (Song et al.; Li et al.).

The two recent original research articles use large -omics molecular data to address answers for clinical problems. The team by Song et al. focuses on the importance of Hsa_circ_000065 in ankylosing spondylitis (AS) and its importance for the intervertebral disc (IVD). Micro RNA (miRNA) research belongs into the most important and fast growing research areas of regenerative medicine and tissue engineering. The field of miRNA is surely one of the most promising research areas for the identification of future therapies (Guo et al., 2023).

The second original research article in this Research Topic then by Li et al. published novel insights on the roles of blood lipid-metabolism genes in immune infiltration using transciptomics data approach could promote the development of intervertebral disc disease (IDD). Both articles use latest state-of-the-art bioinformatics and also provide extensive data on these very important questions.

## 2 Three review articles in the research topic on “hot research topic”

On the side of the review in this Research Topic, we are happy to have received this very detailed study by Li et al. on the description of extracellular vesicles (EVs) and their potential role for IVD repair. This study can be recommended for researchers interested in the fast growing field along with another important review that were recently published on EVs and the field of IVD repair and regeneration (Li et al.; Tilotta et al., 2021).

Furthermore, IL17 has been mainly overlooked in the literature, especially with a special focus on the IVD is then presented by Suyama et al. In this very nice overview review article the reader learns how IL17 might be involved in the autoimmune disease progression in IVD herniation and degeneration. There also seems a correlation between the increase of TH17 T-cell population in patients suffering from disc herniation (Suyama et al.).

The final review that is presented in this Research Topic is written by members of the recently launched “disc4all” consortium. This cross-disciplinary research project, that is funded by a Marie Skłodowska Curie International Training Network (ITN), is at the interface of translational research and consists of computer modelers, biomedical engineers, biologists and clinicians to identify common patterns and strategies that cause the disease in the population. In this review a special focus was done on recent advances in finite element modeling but also in the understanding how immune cells might affect the onset and progress of IVD degeneration (Bermudez-Lekerika et al.).

## 3 Conclusion

This Research Topic is an important hallmark especially highlighting the role of the immune system for IVD research with contributors from wide fields of scientific backgrounds. In the future more articles on translational research on common disabilities that might interfere with IVD-induced low back pain such as diabetes and direct and indirect influence of microbiomes are needed. Furthermore, we will need more studies using–omics approach in the fields of miRNA, transcriptomics, and then meta-studies comparing multiple–omics data with the assistance of artificial intelligence (AI) to help interpretation of the data. Furthermore, EVs might be more applicable as a therapy than injection of cells as a therapy (Li et al.). Overall, we may conclude that this Research Topic is a nice small overview of up-to-date research in the emergent field to identify new cures for IVD-related LBP research.
